# Prevalence and Clinical Impact of Pseudohypercalcemia in Paraproteinemia: A Case and Cohort Study

**DOI:** 10.3390/jcm14217676

**Published:** 2025-10-29

**Authors:** Usman Sunusi, Li Chen, Nianyi Li, Jason K. Y. Lee, Irmeen Siddiqui, Erin Goodhue, Rongrong Huang, Jieli Li

**Affiliations:** 1 School of Dental Medicine, Lake Erie College of Osteopathic Medicine, Bradenton, FL 34211, USA; usunusi@lecom.edu; 2 Department of Pathology, The Ohio State University Wexner Medical Center, Columbus, OH 43210, USA; li.chen@osumc.edu (L.C.); nianyili@gmail.com (N.L.); irmeen.siddiqui@osumc.edu (I.S.); erin.goodhue@osumc.edu (E.G.); 3 Department of Clinical Laboratory, The Ohio State University Wexner Medical Center, Columbus, OH 43210, USA; jason.lee@osumc.edu; 4 Department of Pathology & Immunology, Baylor College of Medicine, Houston, TX 77030, USA; 5 Department of Pathology & Laboratory Services, Harris Health Ben Taub Hospital, Houston, TX 77030, USA

**Keywords:** hypercalcemia, pseudohypercalcemia, paraproteinemia, gamma globulin gap

## Abstract

**Background**: Hypercalcemia is a common and serious complication of malignancy, often contributing to morbidity and mortality. In patients with paraproteinemia, elevated total calcium with normal ionized calcium, termed pseudohypercalcemia, can complicate diagnosis and lead to inappropriate treatment. While this phenomenon has been described in case reports, its prevalence and clinical impact in routine practice remain poorly defined. **Methods**: We report a case of pseudohypercalcemia in a patient with IgG κ multiple myeloma and conducted a retrospective review of de-identified data to assess the prevalence and biochemical associations of pseudohypercalcemia in paraproteinemia. Available data included serum protein electrophoresis (SPEP), total calcium, albumin, total protein, creatinine, and parathyroid hormone (PTH). Associations between calcium status, paraprotein levels, and the gamma globulin gap were examined. **Results**: The index case demonstrated pseudohypercalcemia, with elevated total calcium (13.5 mg/dL) but normal ionized calcium (1.22 mmol/L), in the setting of IgG κ paraproteinemia (4.4 g/dL). In the retrospective cohort of 2537 samples, 986 (39%) had a single monoclonal paraprotein. Gamma globulin gap showed a moderate correlation with paraprotein concentration for IgG (r = 0.56, *p* < 0.0001) and IgA (r = 0.44, *p* < 0.0001), but a weaker relationship for IgM (r = 0.49, *p* < 0.0001). In contrast, total calcium showed no significant correlation with paraprotein concentration in the overall cohort. Among samples with elevated calcium (>10.5 mg/dL), the association between calcium and IgG paraprotein levels remained weak (r = 0.34, *p* = 0.23), and was similar for IgG κ (r = 0.61, *p* = 0.12) and IgG λ (r = 0.09, *p* = 0.87). Hypercalcemia was uncommon, occurring in only ~2% of IgG-positive samples, and rarely at paraprotein levels ≥ 1.5 g/dL. **Conclusions**: Pseudohypercalcemia in paraproteinemia is uncommon but clinically important, as total calcium may be artifactually elevated due to paraprotein-related assay interference, either from assay precipitation effects or calcium binding by paraproteins. Paraprotein burden correlates with gamma globulin gap but not with true calcium status. Reliance on total calcium alone may lead to diagnostic misclassification; ionized calcium should be measured in patients with monoclonal gammopathies to distinguish true hypercalcemia from analytical interference and avoid unnecessary treatment.

## 1. Introduction

Calcium, the fifth most abundant element in the body, is essential for numerous physiological functions, including neuromuscular activity, blood coagulation, and bone metabolism [1]. Measurement of blood calcium is one of the most commonly ordered laboratory tests in clinical practice, serving as a key tool in evaluating calcium homeostasis and diagnosing related disorders [2]. Calcium homeostasis is tightly regulated but can be disrupted by a range of benign and malignant processes, with primary hyperparathyroidism and malignancy together accounting for approximately 90% of hypercalcemia cases [3]. Hypercalcemia of malignancy is the most frequent metabolic complication of cancer, occurring in up to 30% of patients during the course of their disease [4]. It is most often associated with squamous cell carcinomas, breast and renal carcinomas, and hematologic malignancies such as multiple myeloma [5]. Hypercalcemia of malignancy is a marker of poor prognosis and, if untreated, can be life-threatening [6].

The clinical impact of hypercalcemia of malignancy reflects the central role of calcium in physiological function. Disruptions in calcium homeostasis therefore have reflective systemic consequences, as seen in malignancies such as multiple myeloma, where hypercalcemia is a frequent and clinically significant complication [7,8,9]. Multiple myeloma is a clonal plasma cell malignancy and the second most common hematologic cancer in adults [10]. It represents the most frequent malignancy in which the skeleton is the primary site of disease involvement. Hypercalcemia in myeloma arises primarily from extensive tumor-mediated bone resorption, which releases calcium into the extracellular fluid [11]. This process is compounded by the frequent presence of renal dysfunction in myeloma patients, including reduced glomerular filtration and increased tubular calcium reabsorption, which limits the kidney’s ability to excrete the excess calcium load. As a result, serum calcium concentrations can rise markedly. Importantly, the pathogenesis of hypercalcemia in myeloma is multifactorial; not all patients with substantial skeletal involvement develop hypercalcemia, suggesting that additional mechanisms beyond direct bone destruction contribute to its development [12,13,14,15].

While hypercalcemia is a well-recognized complication of multiple myeloma, pseudohypercalcemia is a rare but important diagnostic pitfall. In plasma, calcium exists in three states: free or ionized calcium (~50%), which is the physiologically active form; protein-bound calcium (~40%); and calcium complexed with non-protein anions (~10%) [16]. Ionized calcium is the physiologically active fraction of plasma calcium, directly mediating numerous biological processes. Pseudohypercalcemia is defined by elevated total calcium concentrations with normal ionized calcium levels, meaning patients do not exhibit the typical clinical manifestations of hypercalcemia. In multiple myeloma, pseudohypercalcemia arises from abnormal monoclonal immunoglobulins that directly bind to calcium in circulation, artificially inflating total calcium measurements. The majority of reported cases involve IgG myeloma, with fewer cases linked to IgA myeloma or Waldenstrom’s macroglobulinemia [17,18,19,20,21,22].

Although uncommon, recognizing pseudohypercalcemia is essential to avoid misdiagnosis and inappropriate treatment. Measurement of ionized calcium should be considered in suspected cases, particularly in patients with discordance between laboratory results and clinical presentation. In this study, we report a rare case of pseudohypercalcemia in a patient with multiple myeloma and describe the investigative approach undertaken to confirm the diagnosis. Beyond the case description, we conducted retrospective analyses to further examine the clinical prevalence of such cases and the associations between total calcium concentrations and monoclonal protein (paraprotein) levels or gamma globulin gap in our patient population. This dual approach not only underscores the diagnostic importance of measuring ionized calcium in suspected pseudohypercalcemia cases but also provides insight into how paraprotein levels may influence total calcium measurements. By doing so, we aim to highlight the clinical and laboratory considerations necessary for distinguishing true hypercalcemia from pseudohypercalcemia in multiple myeloma.

## 2. Materials and Methods

The index case was identified during clinical service review and did not require Institutional Review Board (IRB) approval from Baylor College of Medicine, as it involved a single anonymized patient observation. The retrospective data analysis was conducted at The Ohio State University Wexner Medical Center (OSUWMC) and was approved by the OSU Cancer Institutional Review Board (IRB No. 2023C0100).

The index case involved a 54-year-old male who presented with elevated total calcium but normal ionized calcium levels. Laboratory data included total calcium, ionized calcium, albumin, total protein, parathyroid hormone (PTH), parathyroid hormone–related peptide (PTHrP), vitamin D, 1,25-OH vitamin D, serum protein electrophoresis (SPEP), urine protein electrophoresis (uPEP), κ/λ light chain ratio, and immunoglobulin quantification. The diagnosis of multiple myeloma was confirmed by bone marrow biopsy.

This case was used as an index example to showcase the clinical impact and underlying mechanism of pseudohypercalcemia and to inform the design and interpretation of the retrospective cohort analysis. The case was not part of the OSUWMC dataset but served as a representative example linking the clinical findings to the broader analytical investigation, with calcium measurement performed on the same instrumentation platform.

For the retrospective component, de-identified laboratory data were extracted from the OSUWMC for samples tested between January 2021 and December 2021. Samples were included if results were available for serum protein electrophoresis (SPEP), total calcium, albumin, total protein, phosphate, magnesium, alkaline phosphatase (ALP), 25-hydroxyvitamin D [25(OH)D_3_], creatinine, and PTH (when available). To minimize confounding, samples with abnormal creatinine, hypoalbuminemia, or abnormal PTH values were excluded to reduce the likelihood of renal failure or primary or secondary hyperparathyroidism as alternative causes of hypercalcemia.

All biochemical analysis was performed in the OSUWMC clinical laboratory using standardized, accredited methods and instrumentation. Total calcium, albumin, total protein, phosphate, magnesium, and ALP were measured on the Beckman AU series (Beckman Coulter, Indianapolis, IN, USA). Ionized calcium was measured on the Radiometer ABL800 analyzer (Radiometer Medical ApS, Copenhagen, Denmark). Vitamin D was quantified by DiaSorin Liaison XL (DiaSorin., Stillwater, MN, USA). PTH was measured on the Siemens Atellica IM analyzer (Siemens Healthineers USA, Inc., Malvern, PA, USA). Serum protein electrophoresis was performed using the Sebia CAPILLARYS 3 system (Sebia Group, Lisses, France), and paraprotein concentrations were determined by densitometric quantification of M-spike peaks on SPEP.

**Figure 1:**
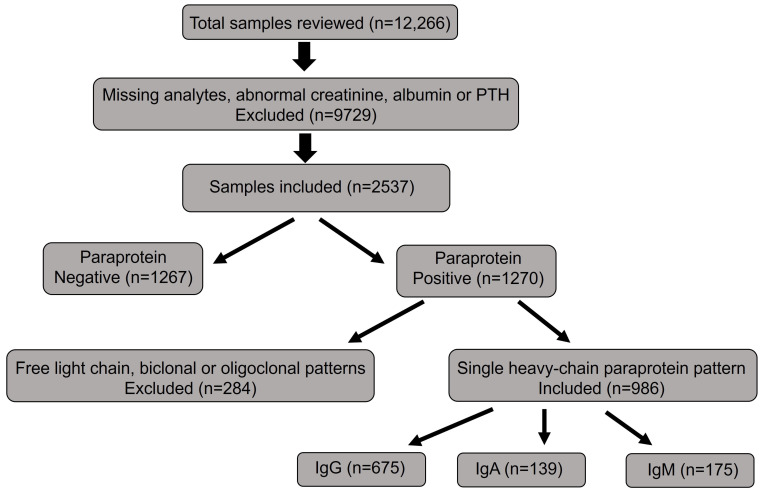
Sample inclusion and stratification flowchart. Flow diagram showing selection of study samples. A total of 2537 de-identified specimens were included; those with missing data or abnormal creatinine, albumin, or PTH values were excluded. Among 1270 paraprotein-positive cases, 986 with a single heavy-chain paraprotein (IgG = 675, IgA = 139, IgM = 175) were retained after excluding samples with free light chain, biclonal, or oligoclonal patterns.

A total of 2537 samples met the inclusion criteria, of which 1267 were negative for paraproteins and 1270 were positive. Among paraprotein-positive samples, 986 with a single heavy-chain paraprotein [IgG (n = 675), IgA (n = 139), and IgM (n = 175)] were included in the final analysis. Samples showing biclonal, oligoclonal, or free light-chain–only patterns were excluded from the study. A flow diagram is used to summarize the number of evaluated and included samples (Figure 1).

For quantitative analysis, abnormal calcium was defined as total calcium greater than 10.5 mg/dL, and an abnormal gamma globulin gap was defined as a gamma globulin gap greater than 4.0 g/dL. Paraprotein concentration was determined by capillary electrophoresis. Samples with incomplete data for any of the measured parameters were excluded.

Associations between continuous variables, including total calcium, gamma globulin gap, and paraprotein concentration, were evaluated using Spearman’s rank correlation analysis in GraphPad Prism (version 10.6.1; GraphPad Software, San Diego, CA, USA). This nonparametric method was chosen because it does not assume data normality or linear relationships. Scatter plots with fitted trend lines were generated to visualize monotonic associations, and Spearman’s correlation coefficients (r) with corresponding *p*-values were reported. A *p* value less than 0.05 was considered statistically significant.

## 3. Results

### 3.1. Case Scenario

Case presentation: A 54-year-old male with past medical history of hypertension and hyperlipidemia presented at the emergency center with chest pain and was found to have a positive treadmill stress test. Cardiac catheterization revealed severe triple-vessel disease requiring coronary artery bypass graft surgery. At admission, he was also noted to have markedly elevated total calcium (13.0 mg/dL; ref 8.6–10.3 mg/dL). Endocrinology evaluations revealed no symptoms of hypercalcemia, kidney stones or fractures. Despite partial improvement with intravenous saline therapy (1000 mL 0.9% NaCl per day for 4 days), total calcium remained above the reference range (to ~10 mg/dL), whereas ionized calcium levels were consistently within the reference range (1.15–1.29 mmol/L) (Table 1). Importantly, the patient exhibited no clinical symptoms of hypercalcemia.

**Figure 2:**
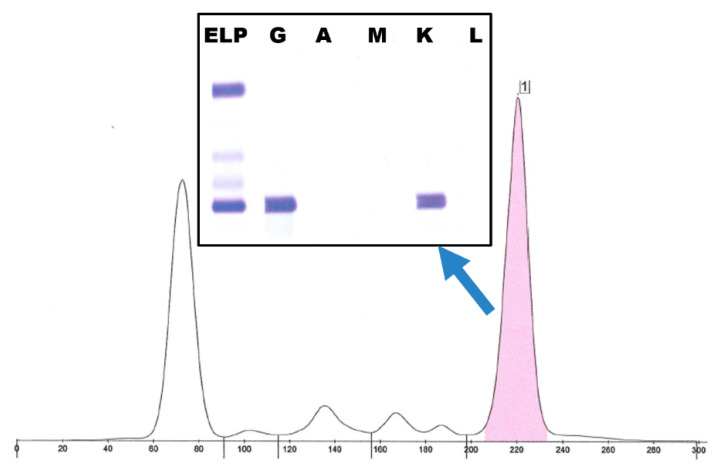
Serum protein electrophoresis and immunofixation showing a distinct IgG κ paraprotein (4.4 g/dL) in a patient with pseudohypercalcemia.

Other laboratory findings: Comprehensive hypercalcemia workup revealed non-suppressed PTH, with high-normal intact PTH of 43.3 pg/mL (ref 8.7–77.1 pg/mL). PTHrP was not detectable. Vitamin D, calcitriol, and 24 h urine calcium were all normal. Albumin was mildly decreased (3.1 g/dL), and total protein was markedly elevated (9.8 g/dL). Serum protein electrophoresis identified an IgG κ monoclonal protein at a concentration of 4.4 g/dL (Figure 2).

While urine protein electrophoresis (UPEP) was normal (no monoclonal protein detected). Immunoglobulin quantification showed elevated IgG (5540 mg/dL) with suppressed IgA (23 mg/dL) and IgM (32 mg/dL); the κ/λ free light chain ratio was normal (1.56) (Table 2).

Final diagnosis: Bone marrow biopsy demonstrated >90% plasma cell infiltration, and skeletal survey revealed multiple lytic lesions, confirming IgG κ multiple myeloma. The discordance between elevated total calcium and normal ionized calcium, in the absence of hypercalcemia symptoms, supported a likely diagnosis of pseudohypercalcemia. This finding suggests a paraprotein concentration-dependent effect on total calcium measurement.

### 3.2. Subtype-Specific Relationships Between Monoclonal Proteins and Gamma Globulin Gap or Hypercalcemia

**Figure 3:**
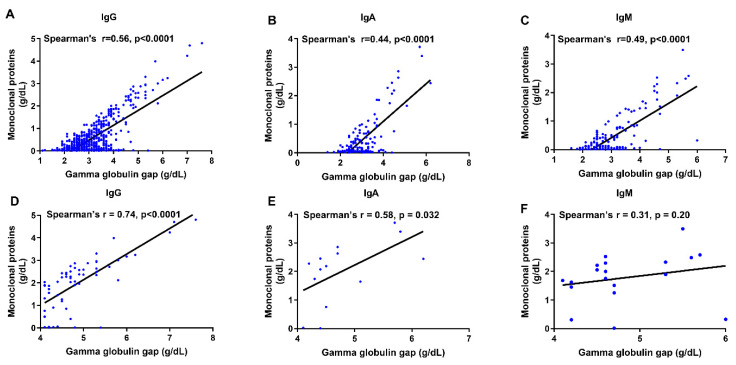
Correlation analyses of paraprotein level and gamma globulin gap in various paraprotein subtypes: (**A**–**C**) Correlation analysis across the entire study group (IgG: n = 675; IgA: n = 139; IgM: n = 175). (**D**–**F**) Correlation analysis on samples with abnormally high gamma globulin gap (>4 g/dL) only (IgG: n = 61; IgA: n = 14; IgM: n = 19).

Correlation analyses demonstrated that the gamma globulin gap was positively associated with monoclonal protein concentration across all paraprotein subtypes. In the overall cohort, moderate correlations were observed for IgG (Spearman r = 0.56, *p* < 0.0001), IgA (r = 0.44, *p* < 0.0001), and IgM (r = 0.49, *p* < 0.0001) (Figure 3A–C). When analysis was restricted to samples with elevated gamma globulin gap (>4 g/dL), the association was strongest for IgG (r = 0.74, *p* < 0.0001) and remained present for IgA (r = 0.58, *p* = 0.032), although interpretation is limited by smaller subgroup size (Figure 3D,E). In contrast, IgM showed a weak and non-significant association in this subgroup (r = 0.31, *p* = 0.20) (Figure 3F). Overall, these findings suggest that the gamma globulin gap reflects paraprotein burden most consistently in IgG paraproteinemia and, to a lesser extent, in IgA paraproteinemia, with limited utility in IgM-associated disease. To avoid overinterpretation, correlations observed in small subgroups, particularly IgM, should be considered exploratory.

**Figure 4:**
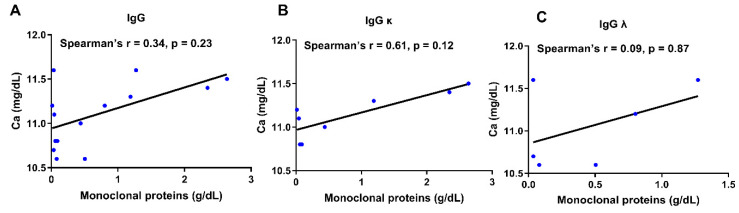
Correlation analyses of paraprotein levels and calcium status in samples with abnormally high calcium levels (Ca > 10.5 mg/dL) for IgG subtype ((**A**) n = 14), IgG Kappa ((**B**) n = 8), and IgG Lambda ((**C**) n = 6).

A separate analysis restricted to samples with elevated total calcium (>10.5 mg/dL) showed no statistically significant correlation between total calcium and IgG paraprotein concentration (Spearman r = 0.34, *p* = 0.23) (Figure 4A). When stratified by IgG subtype, the correlation remained non-significant for both IgG κ (r = 0.61, *p* = 0.12) and IgG λ (r = 0.09, *p* = 0.87) subgroups (Figure 4B,C). Interpretation of these findings is limited by the small number of hypercalcemic cases (n ≈ 14, ~2% of 675 IgG-positive samples), and very few samples in this subset had paraprotein concentrations ≥ 1.5 g/dL. Together, these results indicate that hypercalcemia is uncommon among patients with IgG paraproteinemia, even at higher paraprotein levels. These findings support the hypothesis that when there is a lack of clinical symptoms of hypercalcemia, the elevated total calcium in this population may reflect analytical interference from paraprotein, especially at extremely high levels, rather than true hypercalcemia from multiple myeloma etiology.

## 4. Discussion

In this study, we described an uncommon case of pseudohypercalcemia in a patient with IgG κ multiple myeloma. To further explore the clinical prevalence and impact of such observation, we extended the study to a larger retrospective analysis of patients with paraproteinemia. Our findings demonstrate that although true hypercalcemia is a well-recognized complication of multiple myeloma, elevated total calcium in patients with paraproteinemia may also reflect pseudohypercalcemia, which can arise from either paraprotein–calcium binding or analytical interference from paraproteins, particularly when paraprotein concentrations are relatively high. Importantly, the majority of patients with paraproteinemia did not exhibit elevated calcium, emphasizing that the phenomenon is uncommon but clinically relevant when observed. These results highlight the need for careful interpretation of total calcium values in patients with monoclonal gammopathies and support the diagnostic value of ionized calcium to distinguish true from spurious hypercalcemia [23].

Previous reports of pseudohypercalcemia in multiple myeloma are limited, but most describe cases associated with IgG paraproteinemia, with fewer involving IgA, and rare examples linked to IgM [17,18,19,20,21,22]. Our study broadens this understanding by combining a detailed case with a large retrospective analysis. Our findings are consistent with prior observations that IgG paraproteinemia is most often implicated in pseudohypercalcemia; however, in our cohort, the correlation between paraprotein concentration and total calcium was weak overall and did not reach statistical significance, even within IgG subtypes. Despite this, a trend toward a stronger association was observed in IgG κ compared with IgG λ, suggesting that any effect on total calcium may be subtype-related and concentration dependent rather than universal across paraproteinemia. The underlying mechanism might involve abnormal immunoglobulins binding calcium in circulation, artificially elevating total calcium without affecting ionized calcium [18]. This interpretation is further supported by studies demonstrating that, although albumin is the best-recognized protein affecting the relationship between total and ionized calcium, variations in other serum proteins can also significantly alter this balance [22]. Together, these results provide evidence that pseudohypercalcemia, while uncommon, is a reproducible and paraprotein concentration-dependent phenomenon most likely to occur in patients with high IgG burden.

Among the total of 675 IgG paraprotein samples analyzed, only 2% had elevated calcium, and within this group, very few had paraprotein levels ≥1.5 g/dL. This prevalence is notably lower than reported in prior studies, which estimated hypercalcemia rates of 2.4–21% in paraproteinemia [23,24]. In contrast to our findings, those studies also identified pseudohypercalcemia associated with higher albumin or globulin concentrations, with one recent study reporting a 6.3% prevalence and highlighting hyperglobulinemia ≥ 6.1 g/dL as a significant risk factor [23]. The lower prevalence observed in our cohort may reflect differences in demographics, disease stage, or referral patterns, as well as the inherent limitations of a single-center retrospective study design. Larger multicenter studies with more diverse patient populations will be essential to clarify the true burden of both hypercalcemia and pseudohypercalcemia in paraproteinemia.

An alternative mechanism of the observed pseudohypercalcemia phenomenon is method-dependent photometric interference by paraproteins. Paraproteins are well known to precipitate or increase turbidity under specific reagent conditions, producing false results across multiple analytes in a platform- and chemistry-dependent manner [25,26]. This form of interference is well described for other analytes such as direct bilirubin, HDL cholesterol, and iron, and likely applies to calcium measurement as well. This phenomenon has been repeatedly observed on wet-chemistry analyzers and is often most noticeable in assays that use strongly acidic reaction matrices (for example, Arsenazo III calcium assay as used in our study), where paraproteins can precipitate or scatter light, producing falsely increased total calcium despite a normal ionized calcium, mimicking pseudohypercalcemia. This mechanism is consistent with the broader interference patterns summarized by Yang et al. and Sarkar et al. [25,26]. Thus, in our cohort, the discordance between total and ionized calcium may reflect (i) true binding of calcium by monoclonal immunoglobulins, (ii) assay interference from paraprotein precipitation/turbidity in the Arsenazo III reaction, or (iii) both processes to varying degrees across patients. Laboratory techniques such as inspection of reaction curves, turbidity assessment after reagent mixing, and nonlinear recovery on serial dilution are useful for detecting such interference [25,26]. Because our analysis was retrospective, reaction curve data and dilution studies were not available for confirmation, which we acknowledge as a limitation.

## 5. Conclusions

This study highlights that pseudohypercalcemia, while uncommon, remains a clinically relevant phenomenon that complicates the interpretation of laboratory calcium results. In the overall study population, gamma globulin gap showed a moderate positive correlation with paraprotein concentration, particularly for IgG and IgA monoclonal proteins, supporting its utility as an indirect marker of paraprotein burden. However, total calcium did not correlate with paraprotein concentration in the full cohort. When analyses were restricted to samples with elevated calcium, only weak, non-significant associations were observed, and these did not differ substantially by IgG subtype (κ vs. λ), suggesting that elevated total calcium in paraproteinemia is uncommon and more likely due to paraprotein binding or analytical interference. The discrepancy between our findings and previous reports underscores the need for systematic evaluation across diverse cohorts and various analytical platforms. Clinically, these results emphasize the importance of measuring ionized calcium in suspicious pseudohypercalcemia to avoid misclassification and unnecessary treatment. The findings also support the need for multicenter investigations to better define the prevalence, risk factors, and clinical consequences of both hypercalcemia and pseudohypercalcemia, thereby improving patient management.

## Figures and Tables

**Table 1:** Serial paired total calcium and ionized calcium measurements during hospitalization.

	Day 1	Day 2	Day 3	Day 4	Day 5	Day 6	Day 7	Day 8
tCa (mg/dL)	13.5	12.5	9.8	9.7	9.6	10.4	10.6	10.8
iCa (mmol/L)	1.22	1.23	1.18	1.20	1.19	1.15	1.18	1.23

**Table 2:** Laboratory results in the case scenario.

Test	Results (Reference Range)
Calcium	13.0 mg/dL (8.6–10.3)
Ionized calcium	1.22 mmol/L (1.15–1.29)
Albumin	3.1 g/dL (4.2–5.5)
Total protein	9.8 g/dL (6.0–8.3)
Parathyroid hormone (PTH)	43.3 pg/mL (8.7–77.1)
PTH-related protein (PTHrP)	<2.0 pmol/L (<2.0)
Vitamin D	40.7 ng/mL (30–100)
Calcitriol (1,25-OH vitamin D)	38.5 pg/mL (19.9–79.3)
κ/λ light chain ratio	1.56 (0.26–1.65)
IgG	5540 mg/dL (635–1741)
IgA	23 mg/dL (45–281)
IgM	32 mg/dL (66–433)
IgG κ M-protein	4.4 g/dL

## Data Availability

The data presented in this study are available upon request from the corresponding author.
